# Comparative study of trimming and resection of diseased finger superficial flexor tendons in gouty carpal tunnel syndrome

**DOI:** 10.1186/s12891-023-07050-0

**Published:** 2023-12-02

**Authors:** Jinquan Liu, Fengming Gu, Qianyuan Liu, Wenxuan Chen, Qiuwen Ying, Yi Xu, Aiping Zhu, Li Tang, Danfeng Jing, Zhonghua Xu, Xiaoyun Pan, Jingyi Mi

**Affiliations:** 1 Jintan First People’s Hospital, Changzhou, Jiangsu China; 2 Department of Sports Medicine, Wuxi Ninth People’s Hospital Affiliated to Soochow University, Wuxi, Jiangsu China; 3 Hand surgery department of Ningbo First Hospital, Ningbo, Zhejiang China; 4 Department of Orthopaedic Institute, Wuxi Ninth People’s Hospital Affiliated to Soochow University, NO.999 Liangxi Road, Wuxi City, 214062 Jiangsu Province China

**Keywords:** Gouty carpal tunnel syndrome, Trimming, Resection, Finger function

## Abstract

**Background:**

Hyperuricemia can lead to synovial hyperplasia in the wrist. In severe cases, it can lead to the deposition of gouty stone in the carpal tunnel, resulting in increased pressure in the carpal tunnel and compression of the median nerve to cause carpal tunnel syndrome (CTS), which is called gouty carpal tunnel syndrome (GCTS). As for the surgical treatment of gouty carpal tunnel syndrome, scholars have different opinions on whether it is necessary to remove the superficial flexor tendon. The purpose of this study was to compare the clinical efficacy of trimming and resection of the diseased superficial flexor tendon in the treatment of gouty carpal tunnel syndrome.

**Methods:**

Clinical data were collected from May 2016 to July 2021 from 10 patients (13 affected wrists) diagnosed with gouty carpal tunnel syndrome and classified into two groups according to the surgical modality: the diseased portion of the gout-eroded superficial finger tendon was trimmed in 9 wrists, and the diseased superficial finger flexor tendon was excised in 4 wrists. Values related to flexion and extension functions, 2-PD, DASH, BCTQ, VAS and recurrence in the affected fingers were compared between the two groups as well as before and after surgery in each group.

**Results:**

All affected limbs used were cleared of gouty stones, finger numbness improved, no skin necrosis occurred, and all incisions healed at stage I. At follow-up (13.58 ± 5.53 months), there was no significant difference between groups in flexion and extension function, 2-PD, DASH, BCTQ, and VAS with respect to the affected fingers, and patients in both groups improved significantly before and after surgery. Treatment of only one wrist involved trimming to remove lesion-affected portions of tendon, which reappeared 1 year after surgery, and there was one case of poor recovery from greater piriformis muscle atrophy in both procedures.

**Conclusion:**

Regarding surgical treatment of patients with gouty carpal tunnel syndrome in which the gouty stone has invaded the superficial flexor tendons of the fingers, the diseased superficial flexor tendons can be selectively excised, and the postoperative mobility of the affected fingers may not be impaired.

## Introduction

Carpal tunnel syndrome (CTS) is a common orthopaedic clinical condition with common causes, including degeneration of the carpal bones and joints, hyperplasia, calcification and decreased elasticity of the transverse carpal ligament, and reduced carpal tunnel volume combined with synovial hyperplasia of the carpal joint [1–3]. Recently, scholars have also reported the diagnosis and treatment of cases of carpal tunnel syndrome due to gout [4]. In patients with gout, high uric acid in the blood can cause oedema and synovial hyperplasia in the ligaments and tendons of the wrist. In severe cases, gout stones can be deposited in the carpal tunnel, resulting in a decrease in the volume of the carpal tunnel and an increase in pressure, which can directly or indirectly compress the median nerve [5–7]. We refer to this type of gout-induced carpal tunnel syndrome as gouty carpal tunnel syndrome (GCTS). The incidence of gout-induced carpal tunnel syndrome is very low, and it is still mostly reported on a case-by-case basis. A total of 2684 cases of carpal tunnel syndrome were counted by Rich et al. [4], of which only 15 cases were caused by gout stones in the wrist, accounting for approximately 0.6%. However, in recent years, due to the improvement of quality of life, gouty carpal tunnel syndrome caused by hyperuricaemia is also increasing year by year and becoming more prevalent in younger age groups [8]. The clinical treatment of gouty carpal tunnel syndrome is divided into medical treatment and surgical treatment; medical treatment can control the gout attack and relieve the disease but cannot eliminate the deposited gout stones [7, 9, 10]. Gouty carpal tunnel syndrome can occur with carpal osteonecrosis, rupture of the intercarpal ligament, wrist instability and carpal stiffness. Progression of the disease not only causes irreversible changes in the median nerve but also induces gouty tendonitis and, in severe cases, tendon rupture [11, 12]. Therefore, all carpal tunnel syndrome caused by gout should be treated with early surgery.

However, there are conflicting opinions as to whether the superficial flexor tendon of the finger can be removed for the treatment of gouty carpal tunnel syndrome with gout invasion of the superficial flexor tendon. Some scholars believe that to preserve the postoperative function of the affected finger, it is sufficient to trim the superficial flexor tendon and remove the gout stone deposits in the carpal tunnel without removing the superficial flexor tendon. Other scholars believe that when the gout stone invades the superficial flexor tendon, the superficial flexor tendon can be removed during gout stone removal, and the deep flexor tendon will compensate for the loss of function after surgery [9, 13].

Therefore, we retrospectively analysed the clinical data of 10 patients (13 wrists) with gouty carpal tunnel syndrome who underwent surgical treatment at our hospital between May 2016 and July 2021 to compare the clinical efficacy of both trimming and excision of the diseased superficial finger flexor tendon in the treatment of gouty carpal tunnel syndrome involving the superficial finger flexor tendon.

## Materials and methods

### Study design

This retrospective study was examined and approved by the Institutional Review Committee of our hospital on 07 May 2022 (LW20220036), and the requirement for patients to write/sign an informed consent agreement was waived. All patients who underwent open surgery for gouty carpal tunnel syndrome between May 2016 and July 2021 were identified by review chart and contacted by telephone. All patients were treated by the same doctor at the same medical centre. The baseline demographic data, the duration of symptoms before surgery, and the duration of gout were reviewed.

### Inclusion and exclusion criteria

Patients diagnosed with gouty carpal tunnel syndrome were treated with traditional open surgery at out hospital, and the distal motor latency (DML) of the median nerve of the wrist was ≥ 4.5 ms as detected by electromyography without invasive operation. We excluded patients with a history of wrist trauma or diabetes, polyneuropathy, inflammatory arthropathy, pregnancy, hypothyroidism, malignant tumours, rheumatoid arthritis, alcoholism, infection, secondary carpal tunnel syndrome with tendon sheath cysts or other space-occupying lesions (tumours, hypertrophic synovium, fracture callus and osteophytes) and familial neuropathy. Of course, patients with neurological and mental disorders who were unable to cooperate with treatment were also excluded.

### General information

**Fig. 1:**
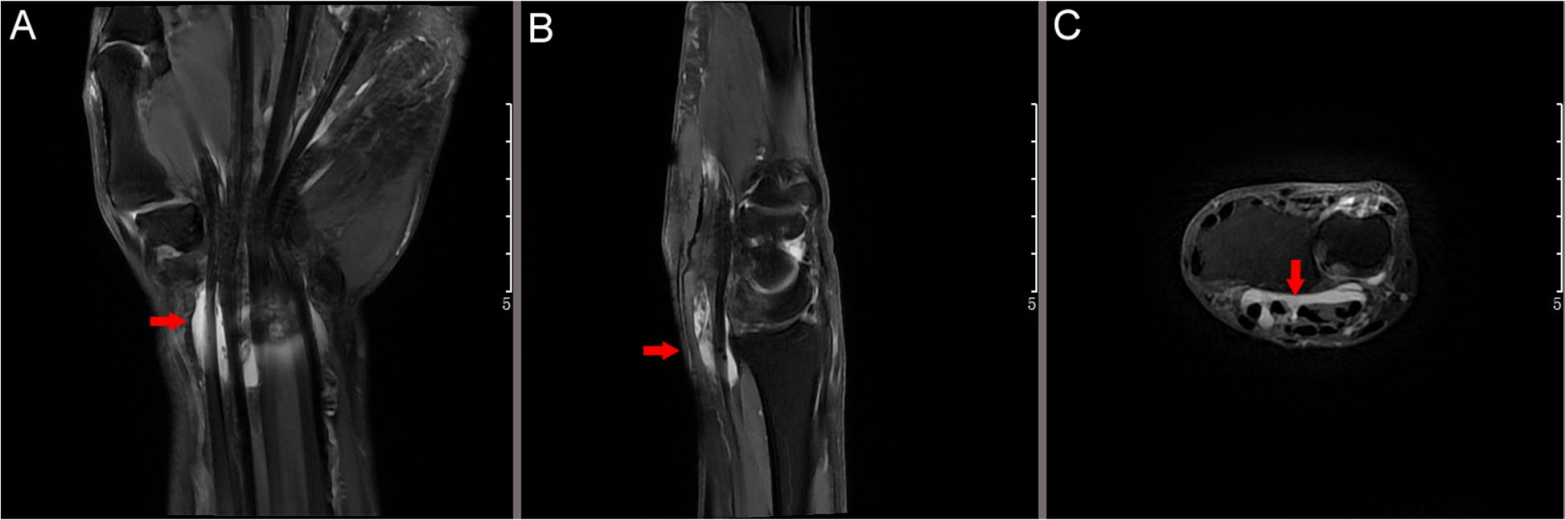
Preoperative magnetic resonance imaging shows a large number of inflammatory synovial hyperplasia in the carpal tunnel, as shown by the red arrow in the image. **A** Positive view, **B** Lateral view, **C** Sagittal position view

A total of 10 male cases (3 left-sided, 4 right-sided, and 3 bilateral) were included, for a total of 13 wrists. Since the onset of gouty carpal tunnel syndrome is rare, each side was considered and counted as one patient in this study. All patients had a preoperative history of gout with a duration of 12.15±7.08 years (5-30 years), none had a recent history of acute phase attacks of gout, and the duration of carpal tunnel syndrome was 2.70±2.91 years (0.33-10 years). The patients all presented with numbness and pain in the three and a half fingers on the radial side, impaired sensation at the fingertips, normal sensation on 3 sides of the greater interosseous muscle group and atrophy on 10 sides. All patients had positive Tinel's sign of the median nerve at the wrist, and all had varying degrees of limitation of finger flexion and extension, with three of them showing limitation of finger extension, ten showing limitation of flexion, and seven having palpable subcutaneous masses on the palmar side of the wrist with slight limitation of wrist flexion and extension (Table 1). Preoperatively, the patients underwent routine MRI to clarify the extent of lesion invasion, which was demonstrated to be within the carpal tunnel (Fig. 1).

**Table 1:** Demographic information of triming and resection patients

	Trim ( *n* = 9)	Resection ( *n* = 4)
Gender, n (%)		
Male	9 (69.2)	4 (30.8)
Age (year) mean ± SE (range)	62.9 ± 1.3 (36–72)	56.0 ± 1.8 (50–64)
BH (cm) mean ± SE (range)	173.1 ± 0.4 (168–180)	172.0 ± 1.4 (165–178)
BW (kg) mean ± SE (range)	69.5 ± 0.5 (63–76)	71.0 ± 1.4 (65–78)
Lesion site, n (%)		
Left	5 (55.6%)	2 (50.0%)
Right	4 (44.4%)	2 (50.0%)
History of gout (year) mean ± SE (range)	12.7 ± 0.9 (5–30)	11.0 ± 1.2 (5–15)
Duration (month) mean ± SE (range)	1.9 ± 0.2 (0.3–6)	4.5 ± 1.1 (0.1–10)
2-PD (mm) mean ± SE (range)		
Thumb	10.8 ± 0.2 (9–15)	11.8 ± 0.5 (10–14)
Index finger	10.7 ± 0.2 (9–14)	10.3 ± 0.5 (9–13)
Middle finger	10.9 ± 0.2 (8–14)	10.8 ± 0.2 (10–112)
Ring finger	10.3 ± 0.2 (8–13)	10.3 ± 0.2 (9–11)
Grip strength (kg) mean ± SE (range)	18.1 ± 0.1 (15–21)	18.8 ± 0.7 (14–23)
Tip pinch strength (kg) mean ± SE (range)	3.9 ± 0.1 (4–5.5)	4.8 ± 0.2 (3–6.5)
BCTQ score mean ± SE		
SSS (range 1–55)	43.1 ± 0.3	43.2 ± 0.9
FSS (range 1–40)	31.1 ± 0.2	32.2 ± 0.4
DASH score (range 0–105) mean ± SE (range)	93.9 ± 0.5 (90–100)	96.5 ± 1.2 (92–102)
VAS (range 1–10) mean ± SE (range)	7.7 ± 0.1 (6–9)	8.5 ± 0.1 (7–9)
Motor function(°)mean ± SE (range)		
Autonomous mobility of the thumb joint	73.9 ± 1.5 (55–90)	70.0 ± 2.7 (55–80)
Autonomous mobility of the finger joints	163.3 ± 2.1 (145–200)	146.3 ± 5.7 (120–160)

*Note*: *SE* standard error, *BH* Body height, *BW* Body weight, *2-PD* Two-Point Discrimination, *DML* Distal Motor Latency, *BCTQ* Boston Carpal Tunnel Questionnaire, *SSS* Symptom Severity Scale, *DASH* Disabilities of the Arm, Shoulder and Hand Questionnaire, *FSS* Functional Status Scale, *VAS* Visual Analogue Score

### Treatment method

**Fig. 2:**
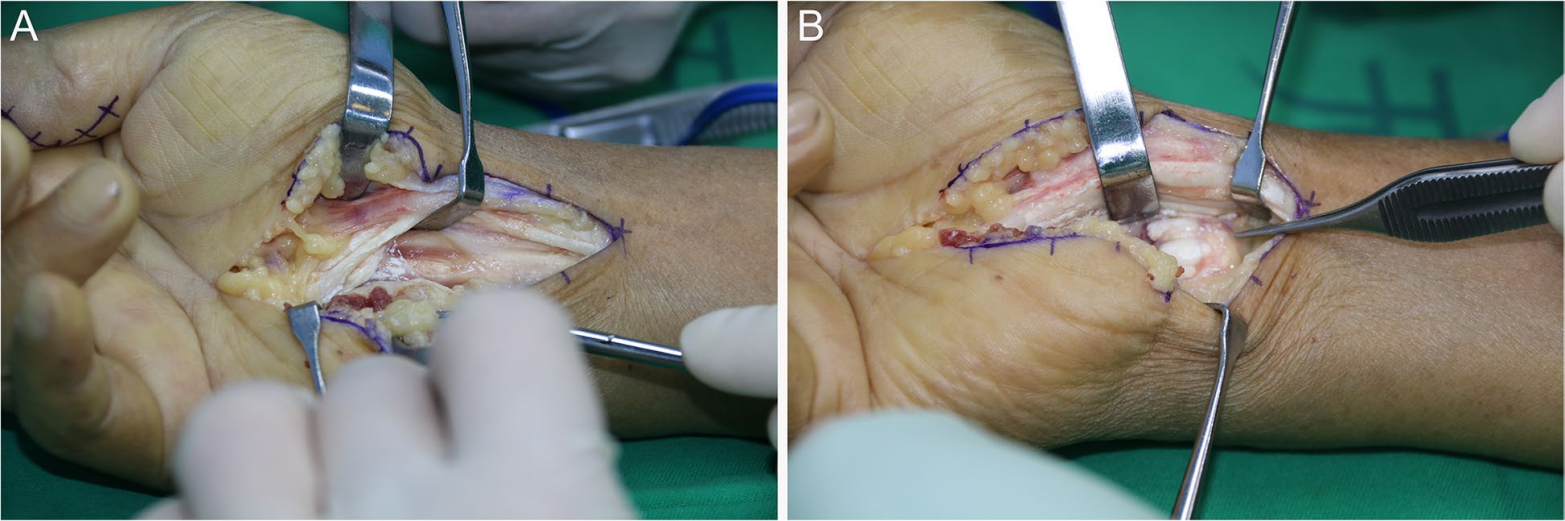
During the operation, gout-like substances were widely distributed in the carpal tunnel. **A** gout eroded the superficial tendon, **B** gout in the carpal tunnel

**Fig. 3:**
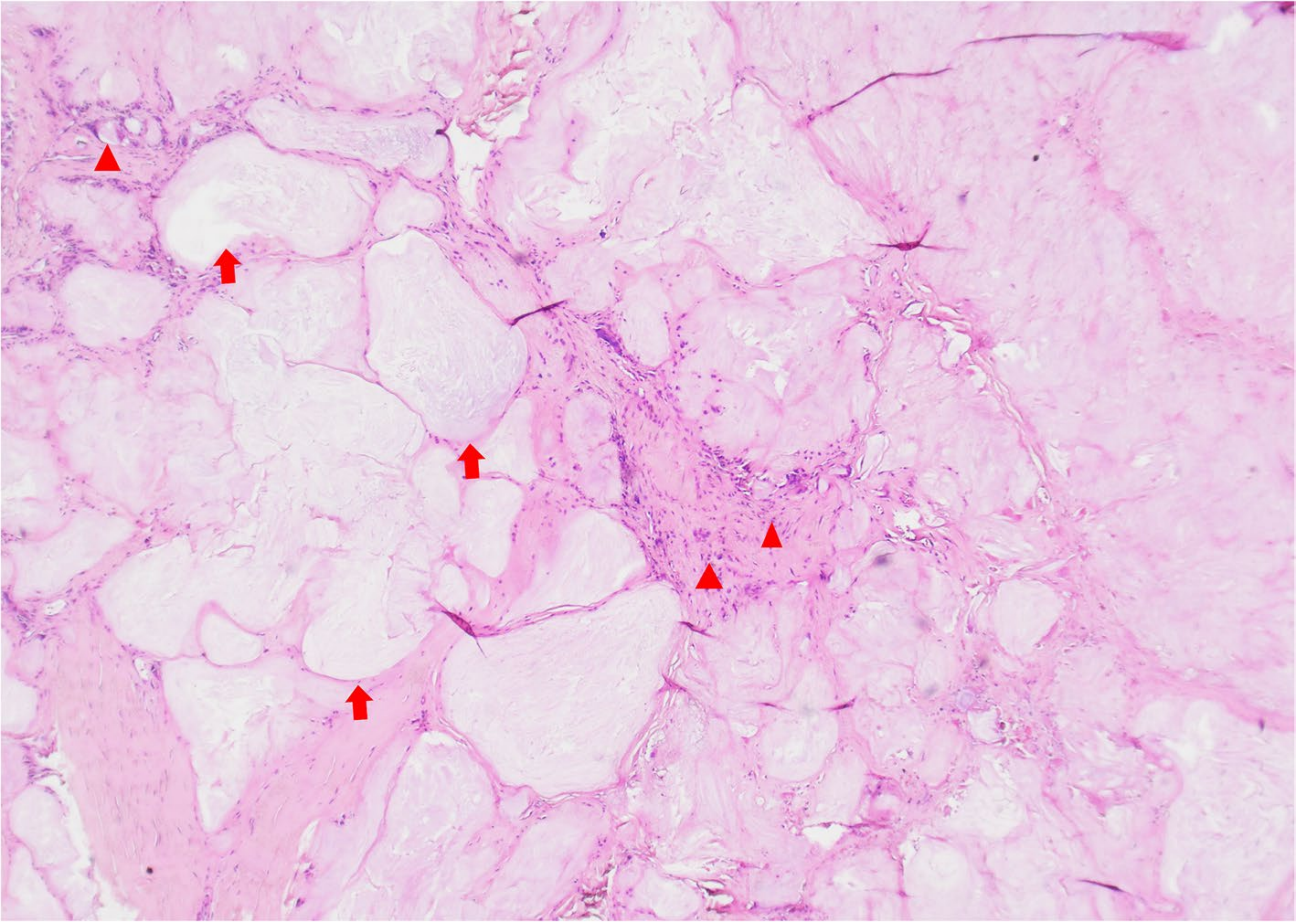
Pathological findings. Gouty nodules, as shown by the red arrow in the image. The red triangle shows the area of inflammation

Under brachial plexus nerve block anaesthesia, the upper middle arm of the affected limb was haemostatically treated with a pneumatic tourniquet at a pressure of 35 kPa. An "S"-shaped surgical incision was made on the surface of the median nerve travel area on the palmar side of the wrist, and the incision was extended distally and proximally as appropriate to the site of gout involvement. During the operation in a typical case, the transverse carpal ligament was incised longitudinally to reveal the median nerve and flexor tendon, and white powdery deposits of different sizes were scattered around the tendon and nerve, partially invading the outer membrane of the median nerve, the tendon, the transverse carpal ligament and the palmar ligament of the wrist. On a total of 7 involved wrists, local indentation of the median nerve and swelling of the proximal nerve were seen (Fig. 2). The gout stone invaded the epineurium and the thickened epineurium, and the stone lesion was removed by epineural release of the median nerve, but the superficial flexor tendon invaded by the gout stone was not excised. The wounds were closed after repeated irrigation with large amounts of saline during the operation. The excised material was sent for pathological examination, and the pathology confirmed the gouty nodules (Fig. 3). Postoperative treatment with uric acid-lowering drugs and early functional exercises for active and passive flexion and extension of each finger were provided.

### Primary measures

Cases that involved trimming of the diseased portion of the superficial flexor tendon of the finger were recorded as the trimming group; cases that involved excision of the diseased superficial flexor tendon of the finger were recorded as the resection group. Data regarding occurrence of incision infection, haematoma formation and recurrence of adverse reactions were collected after surgery; the active and passive flexion and extension mobility of the metacarpophalangeal and interphalangeal joints were recorded to observe their improvement; Disabilities of the Arm, Shoulder and Hand Questionnaire (DASH) score, Boston Carpal Tunnel Questionnaire (BCTQ) score, radial Two-Point Discrimination (2-PD) of the thumb, index, middle and ring finger, pinch and grip strength were determined before and after surgery. Pinch strength, grip strength, two-point discrimination and finger mobility were obtained with the Exacta™ Evaluation Kit Professional (NC-70142-HKP, North Coast Medical, USA). Recovery of flexor and extensor mobility by the thumb and fingers was evaluated by measurement of the total active motion (TAM) of each digit. (TAM = active flexion [metacarpophalangeal (MCP) + proximal interphalangeal (PIP) + distal interphalangeal (DIP)] – active extension deficit [MCP + PIP + DIP]) [14]. After surgery, the researchers asked all patients about the percentage of relief of their hand symptoms (finger pain, sensory abnormalities or CTS-related sensory disorders) when compared to their preoperative condition. At the same time, we also asked the patient whether there was a recurrence.

### Statistical analysis

SPSS 25.0 statistical analysis software (IBM Corporation, USA) was used for statistical analysis of the data in this study. Comparisons of patients' preoperative and postoperative DASH, BCTQ, and Visual Analogue Score (VAS) scores were analysed between The last follow-up visit and preoperative changes (Table 2) using the Wilcoxon two sample rank sum test. A z value >1.96, *p* value < 0.05 was considered statistically significant.

**Table 2:** The last follow-up results of the two methods and the changes of various indexes before operation

Team	Wrists	VAS	DASH	2-PD				BCTQ score	Grip strength (kg)	Tip pinch strength (kg)	Motor function(°)	
				Thumb	Index finger	Middle finger	Ring finger				Thumb joint	Finger joints
Trim	1	7	50	6	7	8	6	30	15.5	8.4	20	70
	2	6	53	5	7	7	6	38	20.4	9.2	18	70
	3	7	45	7	6	6	5	45	16.7	7.6	18	35
	4	8	48	8	5	7	8	40	17.6	6.3	21	25
	5	5	46	6	8	5	6	40	15.4	6.1	17	30
	6	6	52	5	7	6	7	44	18.4	8.6	25	35
	7	5	56	7	7	7	6	37	18.3	6.9	15	45
	8	7	41	8	6	7	6	46	19.4	7.6	18	40
	9	5	39	6	5	6	7	44	19.7	6.8	20	30
Resection	10	7	56	8	7	7	6	38	18.2	7.9	19	45
	11	6	44	7	6	8	7	37	16.5	8.6	25	80
	12	6	50	6	5	7	6	44	17.4	7.1	23	50
	13	5	39	6	8	7	6	40	15.8	6.4	20	60

*Note*: *BCTQ* Boston Carpal Tunnel Questionnaire, *DASH* Disabilities of the Arm, Shoulder and Hand Questionnaire, *VAS* Visual Analogue Score, *2-PD* Two-Point Discrimination

## Results

Ten patients with gouty carpal tunnel syndrome that affected a total of 13 wrists were included in the study, 9 wrists of which were treated with surgical procedures to preserve superficial flexor tendon continuity, with removal of only the gouty stone around the tendon and nerve, and 4 wrists of which were treated with excision of the gouty stone infiltrated superficial flexor tendon of the finger. The results of the last follow-up of the patients treated by the two surgical methods were better than those before operation, and the rank sum test of two independent samples showed that the scores and the improvement of finger function were z < 1.96 and *p* > 0.05. There was no statistical significance between the two surgical methods in the treatment of gouty carpal tunnel syndrome (Table 3).

**Table 3:** The results of the two postoperative changes in Table 2 were tested by the rank sum test of two independent samples

Project		Team	M (P25%, P75%)^a^	95% CI^b^	Wilcoxon two sample rank sum test	
					Z^*^	P^*^
VAS		Trim	6.0 (5.0,7.0)	0.0 (-2.0–1.0)	0.322	0.825
		Resection	6.0 (5.75,6.25)			
DASH		Trim	48.0 (45.00,52.00)	0.5 (-9.0–9.0)	0.232	0.825
		Resection	47.0 (42.75,51.50)			
BCTQ		Trim	40.0 (38.00,44.0)	1.0 (-6.0–7.0)	0.626	0.604
		Resection	39.0 (37.75, 41.0)			
Grip strength, kg		Trim	18.3 (16.7,19.4)	1.05 (-1.1–3.0)	1.080	0.330
		Resection	16.95 (16.32, 17.6)			
Tip pinch strength, kg		Trim	7.6 (6.8,8.4)	-0.15 (-1.6–1.3)	0.232	0.825
		Resection	7.5 (6.925, 8.075)			
Motor function(°)	Thumb joint	Trim	18.0 (18.0,20.0)	-2.5 (-7.0–1.0)	1.484	0.148
		Resection	21.5 (19.75,23.5)			
	Finger joints	Trim	35.0 (30.0,45.0)	-15.0 (-40.0–10.0)	1.784	0.076
		Resection	55.0 (48.25,65.0)			
2-PD	Thumb	Trim	6.0 (6.0,7.0)	0.0 (-2.0–1.0)	0.483	0.710
		Resection	6.5 (6.75,7.25)			
	Index finger	Trim	7.0 (6.0,7.0)	0.0 (-2.0–2.0)	0.080	1.000
		Resection	6.5 (5.75,7.25)			
	Middle finger	Trim	7.0 (6.0,7.0)	-1.0 (-2.0–0.0)	1.438	0.199
		Resection	7.0 (7.0,7.25)			
	Ring finger	Trim	6.0 (6.0,7.0)	0.0 (-1.0–1.0)	0.089	0.940
		Resection	6.0 (6.0,6.25)			

*Note*: *BCTQ* Boston Carpal Tunnel Questionnaire, *DASH* Disabilities of the Arm, Shoulder and Hand Questionnaire, *VAS* Visual Analogue Score, *2-PD* Two-Point Discrimination

^a^M: Median, P25%: First quartile, P75%: Third quartile

^b^95% CI: Median difference

^*^Rank sum test statistics z and p: z > 1.96, *p* < 0.05 considered that there is statistical significance

## Discussion

Different pathologic mechanisms determine the surgical treatment of gouty carpal tunnel syndrome and traditional carpal tunnel syndrome. In gouty carpal tunnel syndrome, the volume of the carpal tunnel decreases due to the occupation of the gouty stone, which causes entrapment of the median nerve in the tunnel and then numbness of the three and a half fingers of the radial side of the hand. In addition, gouty stones in the carpal tunnel often erode the contents of the carpal tunnel, including the median nerve and the superficial and deep flexor tendons of the finger [6]. However, traditional carpal tunnel syndrome often leads to synovial hyperplasia in the carpal tunnel due to inflammation, which reduces the volume of the carpal tunnel but usually does not erode its contents. Based on this, the surgical methods for gouty carpal tunnel syndrome are often different from those for traditional carpal tunnel syndrome. In traditional carpal tunnel syndrome, carpal tunnel incision alone to release the pressure in the carpal tunnel can achieve a good therapeutic effect without excision of the contents to increase carpal tunnel volume [3]. However, for carpal tunnel syndrome caused by the presence of tophi, the tophi in the carpal tunnel often erode the superficial flexor tendon of the finger, and carpal tunnel incision and decompression alone during surgery may lead to recurrence. For example, in this retrospective study, it was found that 1 patient first underwent a simple incision of the transverse carpal ligament to release the median nerve and prune the part of the superficial flexor tendon that was eroded by tophi. When gouty carpal tunnel syndrome recurred after surgery, resection of the shallow flexor tendon of the affected finger was performed at the next visit, and then no recurrence was observed after surgery. Whether this indicates that shallow flexor tendon resection of the finger is less likely to relapse than carpal tunnel incision alone for gouty carpal tunnel syndrome is unclear, and further relevant studies are still needed. In addition, the appropriate length of resection of the superficial flexor tendon in relationship to the extent of gouty carpal tunnel syndrome cannot be recommended in this study due to the small number of patients, and further research is still needed in the future.

In patients with carpal tunnel syndrome caused by gout stone invasion of the superficial flexor tendon, the eroded tendon should be treated accordingly. There are two main treatment methods: In the first method, the eroded superficial flexor tendon of the finger is trimmed, the part eroded by the tophus is removed while preserving continuity, the carpal tunnel is rinsed thoroughly with normal saline, and the tophus in the carpal tunnel is rinsed as much as possible. This method of preserving the continuity of the superficial flexor tendon will not affect the postoperative hand function of the patient. In the second method, the eroded superficial flexor tendon of the finger is excised, and the wrist is rinsed with normal saline. The doctors who choose this method believe that lost postoperative function of the superficial flexor tendon of the affected finger can be compensated by the deep flexor tendon of the affected finger, and there may be no dysfunction of hand activity [9]. Based on the results of this study, the author believes that for carpal tunnel syndrome caused by tophi, selective resection of the superficial flexor tendon of the affected finger will not have a great impact on the postoperative function of the affected finger. Moreover, tendon erosion by tophi is preserved, which seems to leave the risk of carpal tunnel syndrome recurrence because gout disease still exists. It seems that the originally eroded tendons are more likely to accumulate tophi again. Moreover, the original fibrous structure of the eroded superficial flexor tendons is damaged, its strength is weakened, and the possibility of fracture is increased. As previously mentioned, regardless of which surgical method is selected, gout still exists even though the volume of the carpal canal is increased. Postoperative treatment with medical drugs is needed to control the development of gout disease.

Epidemiology and the nature of division of labour cause the disease to be more prevalent in males. Gout attacks are based on abnormal purine metabolism, and men are prone to gout. In social work, men take on more physical labour, and habits of drinking and alcoholism are more common in the male population. As a result, men are more likely to develop gouty carpal tunnel syndrome and have a higher risk of recurrence after surgery [15]. This study included a total of 13 affected wrists in 10 patients, all of whom were male, which is consistent with the epidemiological observation that gout occurs mostly in males. According to the last follow-up results after surgery, all functional scores were significantly improved, and there was no significant difference in postoperative improvement between the two surgical methods. According to the results of the last follow-up, the overall postoperative evaluation and the improvement of finger-joint range of motion also indicated that the two surgical methods had good effect on the treatment of gouty carpal tunnel syndrome, and there was no significant difference in treatment effect between the two.

This study still has some shortcomings. On the one hand, the number of samples is small, and the two groups of samples cannot be matched completely, which is prone to bias. Second, this study is a retrospective study with a large span of study years, a large error rate in follow-up data and a low level of evidence. Prospective cohort studies are still needed in the future to better demonstrate the efficacy of surgical resection of the superficial flexor tendon of the diseased finger.

## Conclusion

In the surgical treatment of gouty carpal tunnel syndrome patients with gout stone invasion and superficial flexor tendon involvement of the finger, selective resection of the superficial flexor tendon of the finger can be performed, and the function of the affected finger may not be impaired after surgery.

## Data Availability

All data generated or analysed during this study are included in this published article.

## Abbreviations

CTS: Carpal Tunnel Syndrome
GCTS: Gouty Carpal Tunnel Syndrome
DML: Distal Motor Latency
2-PD: Two-Point Discrimination
DASH: Disabilities of the Arm, Shoulder and Hand Questionnaire Score
BCTQ: Boston Carpal Tunnel Questionnaire Score
VAS: Visual Analogue Score
TAM: Total Active Motion
MCP: Metacarpophalangeal
PIP: Proximal Interphalangeal
DIP: Distal Interphalangeal

## References

[1] Spinner R, Bachman J, Amadio P (1989). The many faces of carpal tunnel syndrome. Mayo Clin Proc.

[2] Kleopa K. In the Clinic. Carpal Tunnel Syndrome. Annals of internal medicine 163:ITC1. 2015. 10.7326/aitc201509010. 10.7326/AITC201509010 26322711

[3] Padua L, Coraci D, Erra C, Pazzaglia C, Paolasso I, Loreti C, Caliandro P, Hobson-Webb L (2016). Carpal tunnel syndrome: clinical features, diagnosis, and management. Lancet Neurol.

[4] Rich J, Bush D, Lincoski C, Harrington T (2004). Carpal tunnel syndrome due to tophaceous gout. Orthopedics.

[5] Jin M, Yang F, Yang I, Yin Y, Luo J, Wang H, Yang X (2012). Uric acid, hyperuricemia and vascular diseases. Front Biosci (Landmark edition).

[6] Green E, Dilworth J, Levitin P (1977). Tophaceous gout. An unusual cause of bilateral carpal tunnel syndrome. JAMA.

[7] Luo P, Zhang C (2018). Chronic carpal tunnel syndrome caused by covert tophaceous gout: a case report. World J Clin Cases.

[8] Xiao N, Qu J, He S, Huang P, Qiao Y, Li G, Pan T, Sui H, Zhang L (2019). Exploring the therapeutic composition and mechanism of Jiang-Suan-Chu-Bi recipe on gouty arthritis using an integrated approach based on chemical profile, network pharmacology and experimental support using molecular cell biology. Front Pharmacol.

[9] Qiu X, Zhao B, Du X, Jin S, Zhao W (2022). Surgical treatment of hand and foot gout stone and influence factors on Prognosis. Comput Math Methods Med.

[10] Neilson J, Bonnon A, Dickson A, Roddy E (2022). Gout: diagnosis and management-summary of NICE guidance. BMJ (Clinical research ed).

[11] Kim H (2014). Carpal tunnel syndrome caused by tophaceous gout. Korean J Intern Med.

[12] Shin J, Roh H, Chae K, Roh S, Lee N, Yang K (2016). Carpal tunnel syndrome and motor dysfunction caused by tophaceous gout infiltrating 12 tendons. J Clin Rheumatol.

[13] Mockford B, Kincaid R, Mackay I (2003). Carpal tunnel syndrome secondary to intratendinous infiltration by tophaceous gout. Scand J Plast Reconstr Surg Hand Surg.

[14] Mahajan R, Mittal S (2013). Functional outcome of patients undergoing replantation of hand at wrist level-7 year experience. Indian J Plastic Surg.

[15] Dalbeth N, Gosling A, Gaffo A, Abhishek A (2021). Gout. Lancet (London, England).

